# A prospective cohort study to evaluate the incidence of febrile neutropenia in patients receiving pegfilgrastim on-body injector versus other options for prophylaxis of febrile neutropenia: breast cancer subgroup analysis

**DOI:** 10.1007/s00520-022-07025-2

**Published:** 2022-04-14

**Authors:** Reshma L. Mahtani, Rajesh Belani, Jeffrey Crawford, David Dale, Lucy DeCosta, Prasad L. Gawade, Chanh Huynh, Tatiana Lawrence, Sandra Lewis, William W. MacLaughlin, Mohit Narang, Robert Rifkin

**Affiliations:** 1 Miami Cancer Institute, Baptist Health South Florida, Miami, FL USA; 2 Amgen Inc., Thousand Oaks, CA USA; 3 Duke University School of Medicine, Durham, NC USA; 4 Department of Medicine, University of Washington, Seattle, WA USA; 5 Amgen Ltd, Uxbridge, UK; 6 Cancer Care Associates of York, York, PA USA; 7 Riverside Health System, Chesapeake, VA USA; 8 US Oncology Research, Columbia, MD USA; 9 Rocky Mountain Cancer Centers, US Oncology Research, Denver, CO USA

**Keywords:** Pegfilgrastim, Febrile neutropenia, Breast cancer, Compliance, Chemotherapy

## Abstract

**Background:**

Breast cancer chemotherapy often carries a high risk of febrile neutropenia (FN); guidelines recommend prophylaxis with granulocyte colony-stimulating factor (G-CSF), such as pegfilgrastim. Neulasta^®^ Onpro^®^ on-body injector (OBI) is a delivery device administering pegfilgrastim approximately 27 h after application.

**Methods:**

This prospective study examined patients with breast cancer who received chemotherapy with a high risk of FN, receiving OBI (“OBI”) or other options (other G-CSF or none; “other”). The primary endpoint was FN incidence; secondary endpoints included chemotherapy delivery, adherence (G-CSF in all cycles), compliance (G-CSF day after chemotherapy), and FN incidence in patients receiving curative or palliative treatment.

**Results:**

A total of 1776 patients with breast cancer were enrolled (OBI, *n* = 1196; other, *n* = 580). Across all cycles, FN incidence was lower for OBI (4.4% [95% CI, 3.3–5.6%]) than other (7.4% [5.3–9.6%]). For curative treatment, the FN incidence across all cycles was lower for OBI (4.6% [3.4–5.8%]) than for other (7.1% [5.0–9.3%]). For palliative treatment (OBI, *n* = 33; other, *n* = 20), 3 patients (15%) in the other and none in the OBI group had FN. After adjusting for baseline covariates, FN incidence remained lower for OBI (4.6% [3.5–6.1%]) versus other (7.8% [5.7–10.5%]). Adherence was higher for OBI (93.8%) than for other G-CSF (69.8%), as was compliance (90.5 and 53.2%, respectively). Chemotherapy dose delays/reductions were similar for OBI (4.7%/32.3%, respectively) and other (4.7%/30.0%) groups.

**Conclusion:**

Pegfilgrastim OBI was associated with a lower FN incidence in patients with breast cancer compared to other options for FN prophylaxis.

**Trial registration:**

www.clinicaltrials.gov, NCT02178475, registered 30 June, 2014

## Introduction

Febrile neutropenia (FN) is a potentially life-threatening complication that can occur in patients with breast cancer who are receiving myelosuppressive chemotherapy and is associated with significant morbidity, mortality, healthcare resource utilization, and costs [1, 2]. In a recent analysis, the incidence of FN in the USA was as high as 20.6% in patients with breast cancer receiving chemotherapy without prophylaxis for FN [3]. According to real-world data from the USA, the rate of hospitalization for FN in patients with breast cancer was 13.9%, and the mortality rate in hospitalized patients with FN was estimated to be 2.0–2.6%; the mean length of stay ranged from 4.1 to 5.7 days and mean hospital costs ranged from $16,940 to $37,087 USD [1, 2]. Among patients with metastatic breast cancer, resource utilization and mortality rate were even higher: FN-associated hospitalization rate was 89%, and the mortality rate in hospitalized patients was 7.3% [4]. Additionally, FN may necessitate chemotherapy dose delays or reductions, which are significantly associated with increased mortality [5, 6]. Among patients with breast cancer receiving standard myelosuppressive chemotherapy, up to 60% and up to 96% experienced dose delays and dose reductions, respectively [1, 7, 8].

The risk of FN varies based on chemotherapy regimen and comorbidities, and National Comprehensive Cancer Network (NCCN) guidelines recommend prophylaxis with granulocyte colony-stimulating factors (G-CSFs) in patients receiving chemotherapy regimens with a high risk (>20%) of FN and intermediate-risk (10–20%) of FN with ≥1 risk factor for FN [9, 10]. The NCCN recommends following the FDA-approved dosing schedule, which is G-CSF prophylaxis administered the day following chemotherapy [9, 10]. Pegfilgrastim is a long-acting G-CSF that has been shown to reduce the risk of FN by 94% with first-cycle use in patients with breast cancer receiving docetaxel 100 mg/m^2^ every 3 weeks [9, 11]. The Neulasta^®^ Onpro^®^ on-body injector (OBI) is a delivery device that administers pegfilgrastim 6 mg approximately 27 h after application in accordance with NCCN guidelines and FDA recommendations, thus eliminating the need for an office visit the day after chemotherapy administration [12]. In a retrospective study of twenty-eight patients who received chemotherapy and pegfilgrastim OBI between 2016 and 2018, there were no hospitalizations due to FN or chemotherapy dose delays or reductions; however, there was no comparator arm in this study, necessitating additional research [13].

Chemotherapy that carries a high risk of febrile neutropenia is commonly used in breast cancer [14]; however, there is limited evidence of clinical benefits of adherence and compliance to OBI in this patient population. Here, we report analyses of the subgroup of patients with breast cancer with the objective to estimate the incidence of FN in patients with breast cancer who received myelosuppressive chemotherapy and G-CSF prophylaxis with pegfilgrastim OBI or other options; chemotherapy delivery, adherence, and compliance were also evaluated.

## Methods

### Study design and population

The primary analysis of this multicenter, prospective, observational cohort study in patients receiving myelosuppressive chemotherapy and at high risk for developing FN between November 7, 2018, and April 9, 2020, was previously reported [15]. Briefly, in the primary analysis adults were eligible if they had breast, lung, or prostate cancer or NHL, a life expectancy of >6 months, ≥4 planned chemotherapy cycles administered every 3 or 4 weeks, and received a chemotherapy regimen with high FN risk (>20%) or intermediate FN risk (10−20%) and ≥1 risk factor for FN as defined by NCCN guidelines at that time [10]. In this analysis, only patients with breast cancer were included. Patients who received radiation <2 weeks before enrollment or had planned chemotherapy dose reduction for cycle 1, concurrent primary cancers (except non-melanoma skin cancer or adequately treated carcinoma in situ), or significant laboratory abnormalities per the investigator were excluded. Demographic and clinical characteristics, including tumor type, chemotherapy regimen, type of G-CSF prophylaxis received and timing, age, sex, laboratory measurements, comorbidities, and history of other malignancies, were collected. Patients were followed from study enrollment until death, discontinuation of chemotherapy, withdrawal of consent, lose to follow-up, or end of the study.

### Endpoints

The primary endpoint was the incidence of FN during the study period. FN was defined as an absolute neutrophil count (ANC) <1000 × 10^6^/L and occurrence of 1 of the following within 24 h of decreased ANC: temperature >38 °C, use of oral antibiotics (i.e., ciprofloxacin, levofloxacin, moxifloxacin, or amoxicillin-clavulanate), or any intravenous antibiotics. Secondary endpoints included the incidence of FN in patients who received treatment with curative or palliative intent, chemotherapy delivery, adherence, and compliance. Adherence was defined as G-CSF support received in all chemotherapy cycles irrespective of G-CSF administration timing. G-CSF support was defined as the administration of a long-acting G-CSF (pegfilgrastim OBI, pegfilgrastim, or biosimilar pegfilgrastim [pegfilgrastim-jmdb, pegfilgrastim cbqv, pegfilgrastim-bmez]) or ≥10 administrations of a short-acting G-CSF (filgrastim, biosimilar filgrastim [filgrastim-sndz or filgrastim-aafi], tbo-filgrastim, or sargramostim) in each cycle. Compliance with pegfilgrastim was defined as pegfilgrastim OBI, pegfilgrastim, or biosimilar pegfilgrastim administered the day after chemotherapy completion in all cycles in which pegfilgrastim was administered.

### Statistical analysis

Patients with breast cancer were categorized by the type of G-CSF prophylaxis received in the first cycle of chemotherapy: pegfilgrastim OBI (OBI) or other option (other), which included pegfilgrastim pre-filled syringe (PFS), pegfilgrastim biosimilar PFS, filgrastim, tbo-filgrastim, filgrastim-sndz, or no G-CSF prophylaxis selected at the physician’s discretion. Patients remained in the originally-assigned group even if a different type of G-CSF prophylaxis was administered in a subsequent cycle. The incidence of FN was calculated as the percentage of patients who experienced FN, and 95% confidence intervals (CIs) were calculated using a normal approximation method. As reported in the primary analysis, the incidence of FN was adjusted using a standardized log-binomial model to account for confounding variables [15]. These included prior surgery within 6 months before study enrollment, antibiotic use 0-7 days before initiation of chemotherapy, and FN risk of the chemotherapy regimen. Adjusted incidences of FN, relative risks, and associated *p*-values were calculated using a standardized log-binomial model in which weight was assigned to each patient equal to the inverse probability of exposure conditional on that patient’s confounder information [16, 17]. Associated 95% CIs for adjusted incidences and relative risk were calculated using bootstrap methods. Patient characteristics, chemotherapy delivery, adherence, and compliance were summarized using descriptive analyses. Missing data were not imputed.

## Results

### Patient disposition and baseline characteristics

Of the 2347 patients enrolled between November 7, 2018, and April 9, 2020, 1776 had breast cancer (OBI, 1196; other, 580). Baseline characteristics were generally well balanced across groups (Table 1). Patient characteristics were comparable between groups for sex, age, ECOG performance status, number of comorbidities, history of any other malignancy, and prior antibiotic use, chemotherapy, or radiotherapy. A higher percentage of patients in the OBI group had prior surgery within 6 months of enrollment compared with patients in the other group (81.9 versus 69.3%, respectively). Most patients received chemotherapy with curative intent (87.7% in the OBI group; 83.8% in the other group).

**Table 1:** Patient demographics and baseline characteristics

	On-body injector (*N* = 1196)	Other physician choice (*N* = 580)	All patients with breast cancer (*N* = 1776)
Sex, *n* (%)			
Male	3 (0.3)	5 (0.9)	8 (0.5)
Female	1193 (99.7)	575 (99.1)	1768 (99.5)
Age, years			
Median (IQR)	60 (49–68)	58 (48–67)	59 (49–67)
ECOG performance status, *n* (%)			
0–1	1174 (98.2)	574 (99.0)	1748 (98.4)
≥2	14 (1.2)	4 (0.7)	18 (1.0)
Missing	8 (0.7)	2 (0.3)	10 (0.6)
Number of comorbidities, *n* (%)			
>2	173 (14.5)	82 (14.1)	255 (14.4)
≤2	1023 (85.5)	498 (85.9)	1521 (85.6)
History of any other malignancy,^a^ *n* (%)			
Yes	51 (4.3)	34 (5.9)	85 (4.8)
No	1145 (95.7)	546 (94.1)	1691 (95.2)
Antibiotic use 0–7 days prior to initiation of chemotherapy, *n* (%)			
Yes	35 (2.9)	29 (5.0)	64 (3.6)
No	1161 (97.1)	551 (95.0)	1712 (96.4)
Prior surgery,^b^ *n* (%)			
Yes	980 (81.9)	402 (69.3)	1382 (77.8)
No	216 (18.1)	178 (30.7)	394 (22.2)
Prior chemotherapy,^b^ *n* (%)			
Yes	2 (0.2)	0 (0.0)	2 (0.1)
No	1194 (99.8)	580 (100.0)	1774 (99.9)
Prior radiotherapy,^b^ *n* (%)			
Yes	19 (1.6)	10 (1.7)	29 (1.6)
No	1177 (98.4)	570 (98.3)	1747 (98.4)
Intent of treatment, *n* (%)			
Curative	1424 (87.7)	606 (83.8)	2030 (86.5)
Palliative	200 (12.3)	117 (16.2)	317 (13.5)

^a^Excluding nonmelanoma skin cancer. ^b^Within 6 months prior to study enrollment. *ECOG*, Eastern Cooperative Oncology Group; *IQR*, interquartile range

The percentage of patients who received high FN risk chemotherapy regimens was comparable between OBI and other groups (89.4 and 82.2%, respectively; Table 2). The most common chemotherapy regimen with high FN risk was docetaxel and cyclophosphamide (OBI, 47.9%; other, 43.8%) followed by docetaxel, carboplatin, trastuzumab, and pertuzumab (OBI, 32.8%; other, 31.4%). The most common chemotherapy regimen with intermediate FN risk was doxorubicin and cyclophosphamide (OBI, 5.1%; other, 8.1%).

**Table 2:** Baseline febrile neutropenia risk and chemotherapy regimens

	On-body injector (*N* = 1196)	Other physician choice^a^ (*N* = 580)	All patients with breast cancer (*N* = 1776)
FN risk of chemotherapy regimen, *n* (%)			
High	1069 (89.4)	477 (82.2)	1546 (87.0)
Intermediate	127 (10.6)	103 (17.8)	230 (13.0)
Chemotherapy regimen, *n* (%)			
High risk for FN (>20%)			
TC^b^	573 (47.9)	254 (43.8)	827 (46.6)
TCHP^c^	392 (32.8)	182 (31.4)	574 (32.3)
TCH^d^	81 (6.8)	32 (5.5)	113 (6.4)
TAC^e^	23 (1.9)	9 (1.6)	32 (1.8)
Intermediate risk for FN (10–20%)			
AC	61 (5.1)	47 (8.1)	108 (6.1)
AC→T	47 (3.9)	21 (3.6)	68 (3.8)
Docetaxel	8 (0.7)	9 (1.6)	17 (1.0)
TH	5 (0.4)	1 (0.2)	6 (0.3)
Paclitaxel	3 (0.3)	1 (0.2)	4 (0.2)
CMF classic	2 (0.2)	22 (3.8)	24 (1.4)
Bendamustine and rituximab^f^	1 (<0.1)	0	1 (<0.1)
Carboplatin and paclitaxel^g^	0	1 (0.2)	1 (<0.1)
Cisplatin and etoposide^h^	0	1 (0.2)	1 (<0.1)

^a^Other physician choice includes long-acting G-CSF (*n* = 427; pegilgrastrim pre-filled syringe, pegfilgrastim biosimilar pre-filled syringe), short-acting G-CSF (*n* = 45; filgrastim, tbo-filgrastim, filgrastim-sndz), or no G-CSF prophylaxis (*n* = 108); selected at the physician’s discretion. ^b^Docetaxel 75 mg/m^2^ and cyclophosphamide 600 mg/m^2^ every 3 weeks for 4 cycles. ^c^Docetaxel 75 mg/m^2^, carboplatin (AUC 6), trastuzumab (day 1), and pertuzumab (day 1) every 3 weeks for 6 cycles; trastuzumab dosing weekly and every 3 weeks was allowed. ^d^Docetaxel 75 mg/m^2^, carboplatin (AUC 6), and trastuzumab (day 1) every 3 weeks for 6 cycles; trastuzumab dosing weekly and every 3 weeks was allowed. ^e^Docetaxel (75 mg/m^2^ day 1), doxorubicin (50 mg/m^2^ day), and cyclophosphamide (500 mg/m^2^ day 1) every 3 weeks for 6 cycles. ^f^One patient received 4 cycles of bendamustine and rituximab for follicular lymphoma that was incorrectly coded as tumor type “breast” by the site and was included in the analysis. ^g^One patient received carboplatin and paclitaxel, which is not a common chemotherapy regimen for breast cancer. We were unable to obtain clarification from the site regarding the use of this regimen. ^h^One patient received cisplatin and etoposide for poorly differentiated neuroendocrine carcinoma considered to be small cell carcinoma of the breast. *AC*, doxorubicin cyclophosphamide; *AC→T*, doxorubicin, cyclophosphamide→docetaxel; *AUC*, area under the curve; *CMF*, cyclophosphamide, methotrexate, fluorouracil; *FN*, febrile neutropenia; *G-CSF*, granulocyte colony-stimulating factor; *TAC*, docetaxel, doxorubicin, cyclophosphamide; *TC*, docetaxel, cyclophosphamide; *TCH*, docetaxel, carboplatin, trastuzumab; *TCHP*, docetaxel, carboplatin, trastuzumab, pertuzumab; *TH*, docetaxel, trastuzumab

Among patients in the other group during cycle 1, 73.6 and 7.8% of patients received long- and short-acting G-CSF, respectively, and 18.6% received no G-CSF support.

### Incidence of febrile neutropenia

**Fig. 1:**
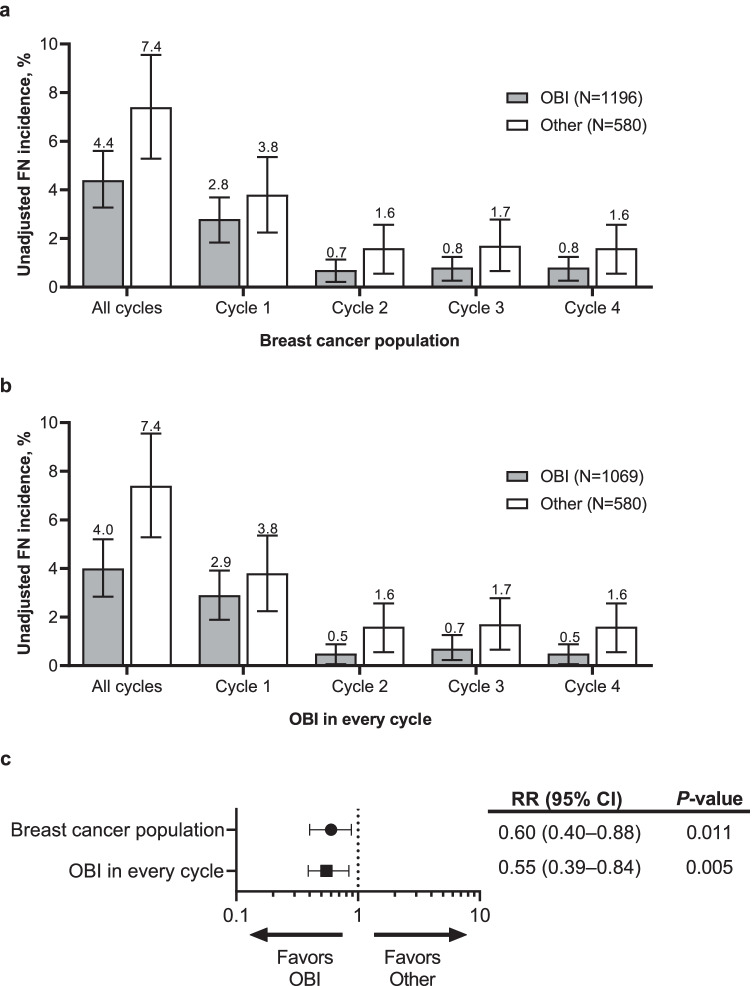
Incidence of FN in patients with breast cancer who received pegfilgrastim OBI or other options. **a** Incidence of FN; percent plus 95% CI. **b** Incidence of FN in patients who received pegfilgrastim OBI in every cycle; percent plus 95% CI. **c** Relative risk of FN. CI, confidence interval; FN, febrile neutropenia; OBI, on-body injector; Other, other physician choice options; RR, relative risk

**Fig. 2:**
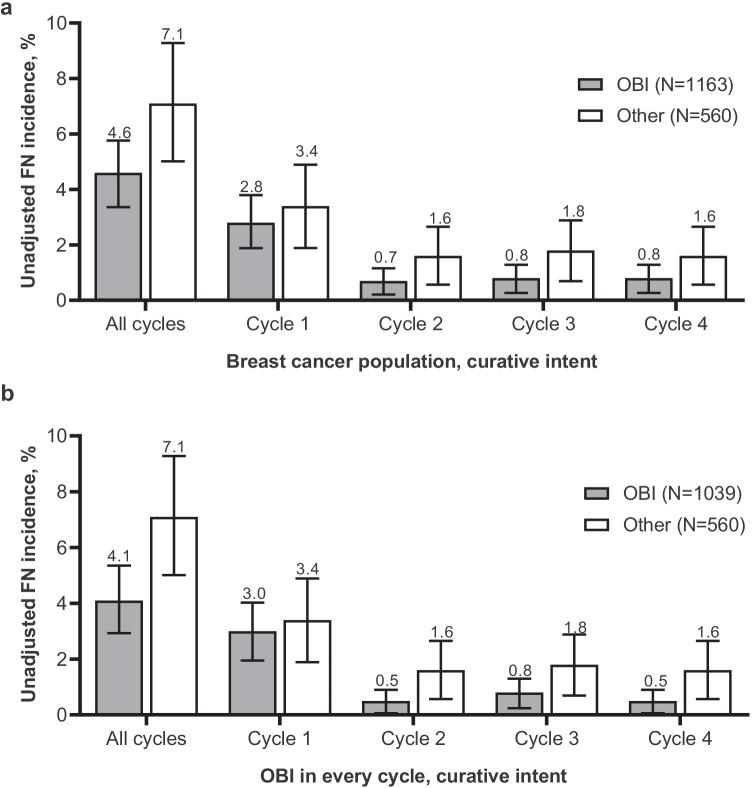
Incidence of FN in patients with breast cancer who received pegfilgrastim OBI or other options with curative intent. **a** Incidence of FN; percent plus 95% CI. **b** Incidence of FN in patients who received pegfilgrastim OBI in every cycle; percent plus 95% CI. CI, confidence interval; FN, febrile neutropenia; OBI, on-body injector; Other, other physician choice options

**Fig. 3:**
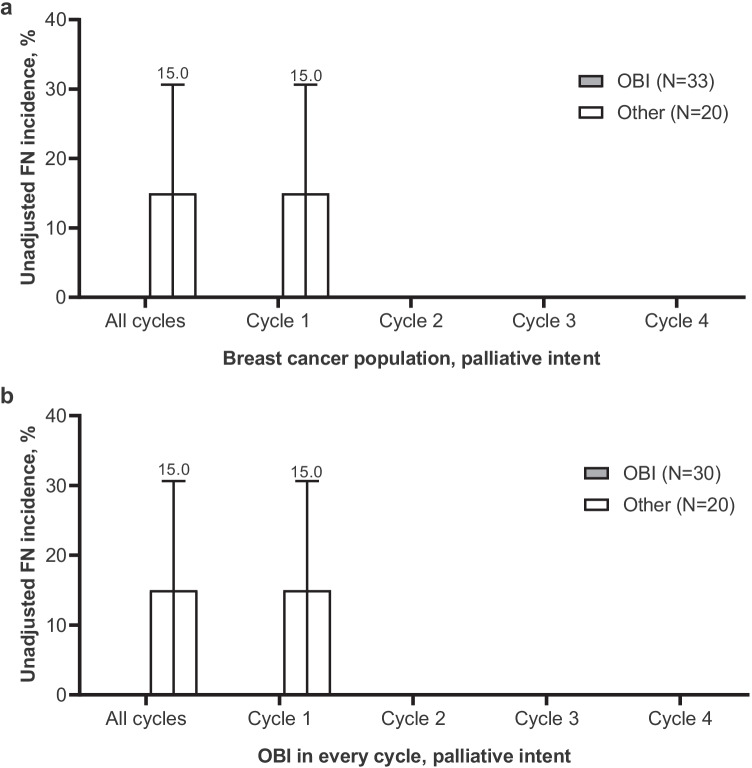
Incidence of FN in patients with breast cancer who received pegfilgrastim OBI or other options with palliative intent. **a** Incidence of FN; percent plus 95% CI. **b** Incidence of FN in patients who received pegfilgrastim OBI in every cycle. The incidence of FN in OBI group was 0% for all cycles. CI, confidence interval; FN, febrile neutropenia; OBI, on-body injector; Other, other physician choice options

Across all cycles, the incidence of FN was lower in patients who received pegfilgrastim OBI (4.4% [95% CI, 3.3–5.6%]) compared with patients who received other options (7.4% [95% CI 5.3–9.6%]; Fig. 1a). The OBI group had a lower incidence of FN at each cycle. Similar trends were observed in patients who received pegfilgrastim OBI in every cycle; across all cycles, the incidence of FN in patients who received pegfilgrastim OBI in every cycle was 4.0% (95% CI, 2.8–5.2%; Fig. 1b). After adjusting for covariates (prior surgery within 6 months prior to study enrollment, antibiotic use 0–7 days prior to initiation of chemotherapy, and FN risk of chemotherapy regimen), the estimated incidence of FN was 4.6% (95% CI, 3.5–6.1%) for the OBI group compared with 7.8% (95% CI, 5.7–10.5%) for the other group. The risk of developing FN was significantly reduced for the OBI group versus the other group (RR, 0.60 [95% CI, 0.40–0.88]; *P* = 0.011; Fig. 1c). The risk of developing FN was further reduced in patients who received pegfilgrastim OBI in every cycle (RR, 0.55 [95% CI, 0.39–0.84]; *P* = 0.005). Similarly, for patients who received treatment with curative intent, the incidence of FN across all cycles was lower in patients who received pegfilgrastim OBI (4.6% [95% CI, 3.4–5.8%]; Fig. 2a) and in patients who received pegfilgrastim OBI in every cycle (4.1% [95% CI, 2.9–5.4%]; Fig. 2b) compared with other options (7.1% [95% CI, 5.0–9.3%]). Of patients treated with palliative intent (OBI, 33; other, 20), FN was only observed during cycle 1 in 3 patients (15%) in the other group (Fig. 3). Two of these patients received docetaxel with no G-CSF in cycle 1; the other patient received docetaxel plus cyclophosphamide plus biosimilar pegfilgrastim in cycle 1.

### Chemotherapy delivery

The percentage of patients who required chemotherapy dose delays or reductions was comparable for patients who received pegfilgrastim OBI or other options. Across all cycles, the percentage of patients with chemotherapy dose delays was 4.7% (95% CI, 3.5–5.9%) for the OBI group and 4.7% (95% CI, 2.9–6.4%) for the other group; at each cycle, the percentage of patients with chemotherapy dose delays was similar between groups. Across all cycles, the percentage of patients with chemotherapy dose reductions was 32.3% (95% CI, 29.6–34.9%) for the OBI group and 30.0% (95% CI, 26.3–33.7%) for the other group, and percentages at each cycle were comparable between groups. Almost all chemotherapy dose delays and reductions occurred after the first cycle.

### Adherence and compliance

**Fig. 4:**
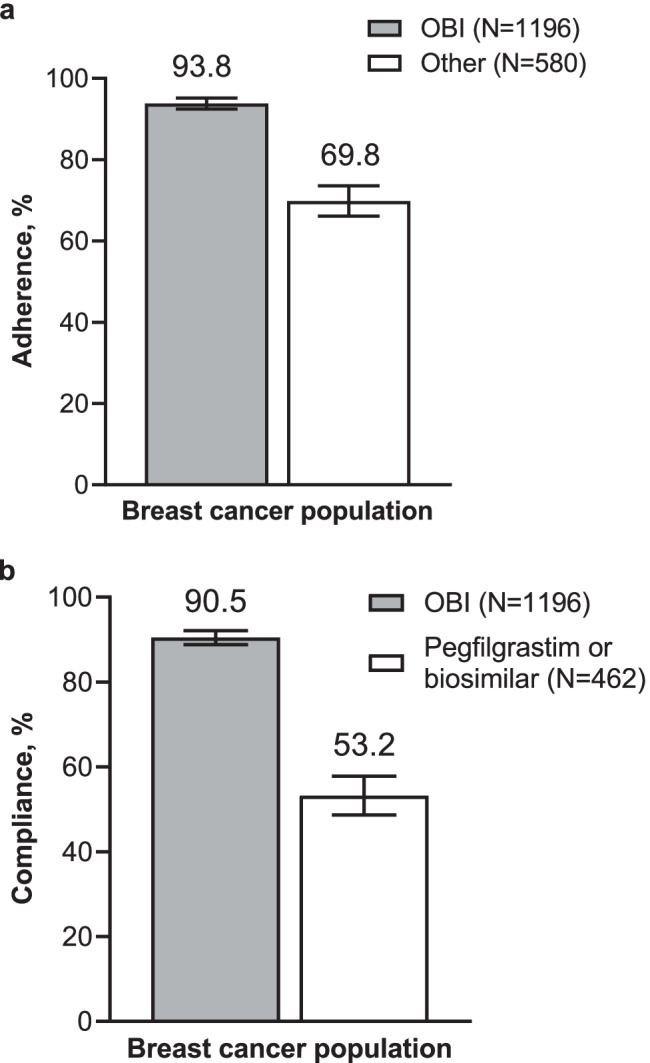
Adherence to G-CSF and compliance to pegfilgrastim in patients with breast cancer who received pegfilgrastim OBI or other options. **a** Adherence; percent plus 95% CI. **b** Compliance; percent plus 95% CI. G-CSF, granulocyte colony-stimulating factor; OBI, on-body injector; Other, other physician choice options

Adherence to G-CSF (1 long-acting G-CSF or 10 short-acting G-CSF per chemotherapy cycle, regardless of timing) was higher in patients who received pegfilgrastim OBI (93.8% [95% CI, 92.5–95.2%]) compared with patients who received other options (69.8% [95% CI, 66.1–73.6%]; Fig. 4a). Of patients who received pegfilgrastim, compliance (a receipt of long-acting G-CSF the day after the last day of chemotherapy) was higher in patients in the pegfilgrastim OBI group (90.5% [95% CI, 88.8–92.1%]) compared with patients in the other group (*n* = 462; 53.2% CI [48.7–57.8%]; Fig. 4b).

## Discussion

This sub-analysis of a prospective observational study evaluated clinical outcomes in patients with breast cancer who received myelosuppressive chemotherapy and G-CSF support as pegfilgrastim OBI or other options for prophylaxis of FN. The overall incidence of FN was lower in patients who received pegfilgrastim OBI in the first chemotherapy cycle compared with other options, regardless of the use of pegfilgrastim OBI in subsequent cycles. The risk of developing FN was reduced by 40% with pegfilgrastim OBI compared with other options. The incidence of dose delays and reductions was similar among patients who received pegfilgrastim OBI or other options. Adherence with G-CSF prophylaxis and compliance to pegfilgrastim were higher in patients who received pegfilgrastim OBI compared with patients who received other options.

Our findings are consistent with retrospective studies of patients receiving myelosuppressive chemotherapy for non-metastatic solid tumors or NHL. In a study of patients with non-metastatic breast cancer or NHL receiving high or intermediate FN risk chemotherapy regimens, the incidence of FN in the first cycle was 4–9% in patients who received G-CSF prophylaxis [18]. In another retrospective study of patients with non-metastatic solid tumors (including breast) or NHL receiving high or intermediate FN risk chemotherapy regimens, the incidence of FN in the first cycle ranged from 2.2–3.8% in patients who received G-CSF prophylaxis [19]. The incidence of FN reported in a retrospective study of patients with metastatic breast cancer was higher than observed in the present study; the overall incidence of FN was 15.8%, and the rate of G-CSF prophylaxis was 16.7% in this population [4]. However, treatment in the metastatic setting is typically palliative; the American Society of Clinical Oncology guidelines did not find the demonstrable benefit of G-CSF prophylaxis in patients with metastatic disease, which is consistent with the small number of patients who received G-CSF prophylaxis in the palliative intent subgroup of the present study [6]. In this study, the risk of FN in patients receiving “other” treatment with palliative intent appears to be twofold higher for the patients receiving “other” treatment with curative intent; however, the small patient numbers in these groups mean these data should be interpreted with caution.

The decreased incidence of FN observed with pegfilgrastim OBI in this study may be associated with increased adherence and compliance in this group relative to patients who received other options [20]. Adherence was 34.4% higher, and compliance was 70.1% higher in patients who received pegfilgrastim OBI compared with patients who received other options.

In prospective and retrospective studies, receipt of pegfilgrastim at least 24 h after myelosuppressive chemotherapy administration resulted in improved adherence and patient outcomes [15, 21, 22]. Pegfilgrastim OBI allows for next-day administration without a clinic visit, therefore reducing the patient travel burden, reducing noncompliance because of the travel burden, and optimizing outcomes [23, 24].

### Limitations

The definition of FN used in this study uses an ANC that may be higher than that used to define FN in some clinical settings [25]. However, as the definition was used across both groups, it is not expected to affect the generalizability of the results. Selection bias may exist because of patients being lost to follow-up after enrollment (OBI group, *n* = 16; 1.3%; other group, *n* = 10; 1.7%), and therefore it would not be possible to evaluate the risk of FN among these patients. Additionally, these results may be confounded by indication because patients who were perceived to be at higher risk by the investigator were more likely to receive G-CSF prophylaxis. In the analysis of adherence, patients with a better prognosis or lower ECOG may have been prescribed fewer doses of short-acting G-CSF prophylaxis and, thus, may have been inappropriately classified as non-adherent. In addition, reasons for lack of adherence were not captured to the extent required for further analysis. Reasons for lack of compliance were not collected. Patients receiving same-day G-CSF could have been considered noncompliant; however, the percentage of patients receiving same-day G-CSF in the overall study population (~1%; *n* = 28/2575) was small enough that any difference in the incidence of FN in this subgroup would be unlikely to bias the study results. Factors associated with possible sources of confounding by indication were included in the standardized log-binomial regression, but bias can result from unmeasured and unknown risk factors. Finally, the study closed earlier than planned due to the COVID-19 pandemic, and the target sample size was not reached, thus it was not possible to compare patients with curative versus palliative treatment intent.

## Conclusions

Patients with breast cancer at high risk of developing FN who received pegfilgrastim OBI had a lower incidence of FN compared with patients who received other options. The decreased incidence of FN in the OBI group may be a result of increased adherence and compliance to G-CSF prophylaxis compared with other options.

## Data Availability

Qualified researchers may request data from Amgen clinical studies. Complete details are available at http://www.amgen.com/datasharing.
